# Target-similarity search using *Plasmodium falciparum* proteome identifies approved drugs with anti-malarial activity and their possible targets

**DOI:** 10.1371/journal.pone.0186364

**Published:** 2017-10-31

**Authors:** Reagan M. Mogire, Hoseah M. Akala, Rosaline W. Macharia, Dennis W. Juma, Agnes C. Cheruiyot, Ben Andagalu, Mathew L. Brown, Hany A. El-Shemy, Steven G. Nyanjom

**Affiliations:** 1 Department of Molecular Biology and Biotechnology, Pan African University Institute of Science, Technology and Innovation, Nairobi, Kenya; 2 Department of Emerging Infectious Diseases (DEID), United States Army Medical Research Directorate-Kenya (USAMRD-K), Kenya Medical Research Institute (KEMRI)—Walter Reed Project, Kisumu, Kenya; 3 Centre for Biotechnology and Bioinformatics, University of Nairobi, Nairobi, Kenya; 4 Department of Biochemistry, Faculty of Agriculture, Cairo University, Giza, Egypt; 5 Department of Biochemistry, Jomo Kenyatta University of Agriculture and Technology, Nairobi, Kenya; University of Melbourne, AUSTRALIA

## Abstract

Malaria causes about half a million deaths annually, with *Plasmodium falciparum* being responsible for 90% of all the cases. Recent reports on artemisinin resistance in Southeast Asia warrant urgent discovery of novel drugs for the treatment of malaria. However, most bioactive compounds fail to progress to treatments due to safety concerns. Drug repositioning offers an alternative strategy where drugs that have already been approved as safe for other diseases could be used to treat malaria. This study screened approved drugs for antimalarial activity using an *in silico* chemogenomics approach prior to *in vitro* verification. All the *P*. *falciparum* proteins sequences available in NCBI RefSeq were mined and used to perform a similarity search against DrugBank, TTD and STITCH databases to identify similar putative drug targets. Druggability indices of the potential *P*. *falciparum* drug targets were obtained from TDR targets database. Functional amino acid residues of the drug targets were determined using ConSurf server which was used to fine tune the similarity search. This study predicted 133 approved drugs that could target 34 *P*. *falciparum* proteins. A literature search done at PubMed and Google Scholar showed 105 out of the 133 drugs to have been previously tested against malaria, with most showing activity. For further validation, drug susceptibility assays using SYBR Green I method were done on a representative group of 10 predicted drugs, eight of which did show activity against *P*. *falciparum* 3D7 clone. Seven had IC_50_ values ranging from 1 μM to 50 μM. This study also suggests drug-target association and hence possible mechanisms of action of drugs that did show antiplasmodial activity. The study results validate the use of proteome-wide target similarity approach in identifying approved drugs with activity against *P*. *falciparum* and could be adapted for other pathogens.

## Introduction

Malaria is an infectious disease with high morbidity and mortality. Approximately 3.3 billion people are at risk of getting malaria [1]. In 2015 alone, there were an estimated 212 million new cases of malaria worldwide with about 429,000 deaths reported [2]. Out of the total reported malaria cases and deaths, 90% of them occur in Africa, followed by the South-East Asia [1]. This disease burden is aggravated further by rapid development of resistance to antimalarial drugs. Reports of resistance to artemisinin-based combination therapy (ACT), the recommended first-line treatment for *Plasmodium falciparum* malaria [3–4] in Southeast Asia [5] warrants urgent discovery of new antimalarial drugs.

There are several drug discovery methods that have been used in malaria research [6]. Most approaches involve the use of either target-based or whole cell-based high throughput screens [7–11]. In target-based approaches, extracted proteins that are crucial for the parasite survival are assayed against huge compound libraries, a strategy that was used in the discovery of inhibitors of *P*. *falciparum* dihydroorotate dehydrogenase [12]. On the other hand, the whole cell-based approach involves exposing the *P*. *falciparum* parasite to test compounds to determine their inhibitory activities. Some antimalarial drugs have been modified from already existing drugs, these include synthetic ozonides which are based on artemisinins [13]. Modifications of drug compounds during drug development is done to either optimize their therapeutic activities, counteract the effect of resistance to the scaffold drug or mitigate the drug’s side effects. Many effective antimalarial drugs have been derived from traditionally used herbal medicines [6], this includes quinine which is extracted from the *Cinchona* trees and artemisinins are got from the Chinese herb *Artemisia annua* [14].

Use of Computer Aided Drug Discovery and Development (CADDD) to complement traditional approaches has greatly reduced cost, time and risks in chemotherapy research [15]. CADDD has successfully been used in the discovery of several drugs that have either been approved or are in clinical trials [16]. *In silico* tools that have been used in drug discovery and development can be broadly classified into bio-chemical databases, chemoinformatics and tools used in structure-based and ligand-based drug design [17].

The effectiveness of an antimalarial drug is dependent on its ability to target a protein or a biological pathway that is essential for the survival of the parasite in the blood stages. The shift of intervention strategies towards pre-elimination in some parts of the world has motivated targeting of other stages of the parasite [9,18–21]. The completion and annotation of the *P*. *falciparum* genome [22,23] revealed metabolic pathways that are essential in various stages of the parasite. For instance, heme biosynthesis is essential for *P*. *falciparum* in mosquito stage but not in asexual blood stages [24]. Drugs targeting this pathway are unlikely to provide successful antimalarial treatment but may be useful as transmission blockers. Similarly, type II fatty acid biosynthesis is essential for sporozoite development in the mosquito but not in the erythrocytic stages [25]. Tricarboxylic acid (TCA) cycle is nonessential in asexual parasites but has been shown to be indispensable in transmission stages of *P*. *falciparum* [26]. Pathways that are crucial in all stages of parasite development include phospholipid biosynthesis [27], coenzyme folate biosynthesis [28], glycolysis [29] and pentose phosphate pathway [30]. Several enzymes and other proteins classes involved in these pathways have been investigated as potential drug targets, with proteases [31–36] and kinases [37–42] being some of the most studied.

Development of new drugs to the point of their introduction into the market is expensive and time-consuming, costing about $100–800 million over a period of 12–15 years [43]. Moreover, most drugs that show activity against malaria fail to get approved due to safety concerns. Consequently, some drug discovery strategies have focused on drug repositioning which entails using already existing drugs for indications different from those they were approved for in order to circumvent approval challenges [44]. This approach has been widely explored in malaria chemotherapy research [45–50].

Through a target-similarity approach, this study sought to predict approved drugs that have undiscovered activity against *P*. *falciparum* and hence could be repositioned as antimalarials. This is based on the principle that a drug would have a similar effect on a protein that is similar to its putative target. Two previous studies have used a similar approach in repositioning of approved drugs against *P*. *falciparum* apicoplast [51] and *Schistosoma mansoni* [52]. In this study, each *P*. *falciparum* protein sequence in NCBI RefSeq database was used to search for similar to putative drug targets. Functional regions of the successful drugs targets were determined and used to fine tune the similarity search. This study identified approved drugs that have antimalarial activity and possible *P*. *falciparum* proteins they could be targeting.

## Material and methods

### Mining of *P*. *falciparum* proteome

A list of all proteins expressed in all stages of *P*. *falciparum* was obtained from NCBI Reference Sequence (RefSeq) database release 75 [53]. RefSeq database was preferred over GenBank because of the non-redundant nature of its sequences and the fact that it provides the best available sequence in GenBank (reference sequence) for a each protein. The search at NCBI was made by use of key words “*Plasmodium falciparum*” and selecting “Protein” database before initiating the search. To further filter the results, “*Plasmodium falciparum*” was selected in the organisms section and RefSeq as the source database. All the protein sequences were downloaded in a single multi-FASTA file. For easy manipulation, the downloaded sequences were converted into a CSV spreadsheet using R statistical programming software [54].

### Identification of putative drug targets using drug databases

Using each *P*. *falciparum* protein sequence as a query, a search was done for similar putative drug targets on three publicly available databases; DrugBank [55], Therapeutic Target Database (TTD) [56] and STITCH 4.0 [57]. These databases have information on drugs, their putative targets and other drug-related information. Homologous proteins with output expectation values (E values) lower than 1e-20 [52] were considered for further analysis while the rest were excluded. Here, the E value describes the number of times one can expect to see a match by chance, thus the lower the E value the better. The putative drug targets that met the similarity threshold were retrieved with approved drugs that target them and keyed into a spreadsheet alongside their homologous *P*. *falciparum* proteins. For the STITCH database, drugs and other biomolecules that interact with the *P*. *falciparum* proteins are already predetermined. Therefore, a search was made in the STITCH database for each of the *P*. *falciparum* proteins, drugs that are predicted to interact with the proteins with a confidence score of at least 0.7 were considered for further analysis.

### Determination of druggability index

The druggability indices for all the predicted *P*. *falciparum* target proteins was obtained from TDR Targets Database v5 [58,59]. Druggability index (D index) describes how druggable a protein is, that is how likely the protein is modulated with a small molecule drug [60]. Druggability indices range from 0 (least druggable) to 1.0 (most druggable). These scores reflect a number of factors such as how similar the protein is to a library of targets at ChEMBL database [61], whether the protein has physiochemical features of known drug targets and empirically determined interactions with drug like compounds. This step was important in resolving viable *P*. *falciparum* drug targets. If a drug is predicted to target a protein with low druggability but still exhibits high antimalarial activity then that could imply the drug inhibits another protein with high druggability.

### Determination and comparison of functional amino acid residues

All the *P*. *falciparum* protein targets that met the inclusion criteria from the drug database search were then analyzed to determine if they share functional amino acid residues with their homologous putative drug targets. Amino acids residues that are conserved by evolution in a protein are believed to perform important structural and/or functional roles in the protein. The previous similarity search in drug databases weighted all amino acids equally while this step only checked for similarity in conserved amino acid residues. This is important in fine tuning the search because two proteins would be more likely to share ligands if they shared functional and structural regions. Before determining the functional residues, a protein-protein pairwise alignment using BLAST [62] was done at NCBI with the drug target as the query sequence and its corresponding *P*. *falciparum* homolog as the subject. Only proteins pairs that had more than 80% query coverage were considered for ConSurf server analysis. This step ensured that only proteins that are likely to share a significant number of residues proceeded to analysis using the ConSurf server.

**Fig 1:**
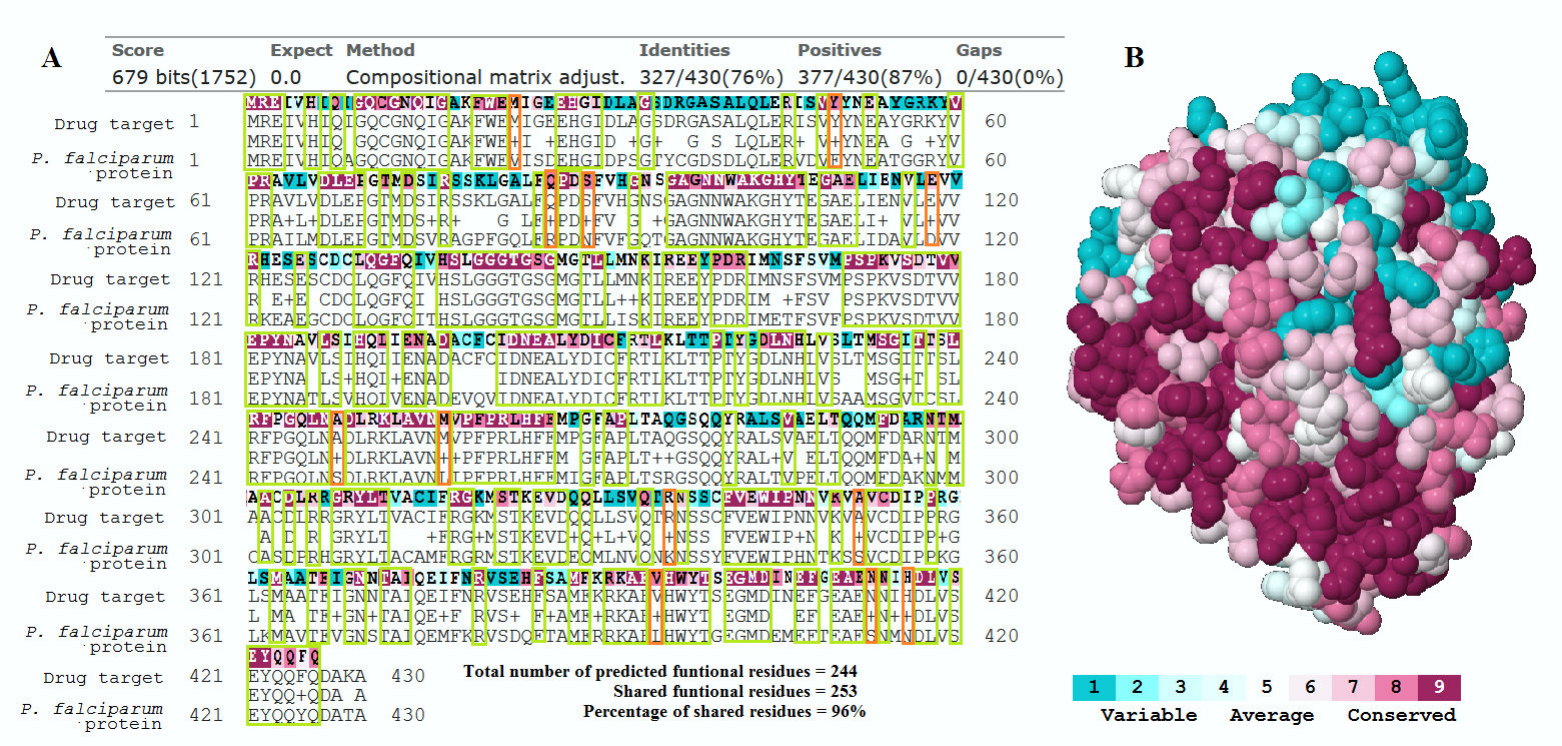
Comparison of conserved amino acid residues. (A) ConSurf server MSA results (color coded according to conservation scores) of the drug target, the human tubulin beta-1 (NCBI accession number NP_110400.1) is overlaid above its BLAST pair-wise alignment with its *P*. *falciparum* homolog (NCBI accession number XP_001347369.1). The percent of the shared conserved residues was then determined; (B) 3D molecular structure of the human tubulin beta-1 chain with residues color coded according to their conservations scores, this was part of the ConSurf server results.

The ConSurf server [63] determines functional amino acid residues by estimating the degree of conservation of amino acids across 150 close sequence homologues obtained from UniProt database [64]. Evolutionary conservation of amino acid positions is estimated based on the phylogenetic relationship between the homologous sequences which is determined by neighbor joining approach with maximum likelihood distance. Conservation scores are calculated using the Bayesian method. The spatial orientation of the amino acids in the 3-D structures of the proteins are also considered in the ConSurf algorithms hence the requirement to have the protein sequence inputs in a PDB format. The 3-D structures were either obtained from Protein Data Bank in Europe [65] or modelled using SWISS-MODEL server [66] if they were not available in the PDB database. The Consurf server result outputs included multiple sequence alignment (MSA) with the amino acid residues color coded according to their conservation scores. The MSA was snipped using Windows^®^ “snipping tool” and overlaid over the BLAST protein-protein pairwise alignment results as shown in Fig 1. This aided in visual comparison and determination of conserved amino acid residues that are shared with the corresponding homologous *P*. *falciparum* protein. Amino acid residues with conservation scores of at least six and are shared between the two proteins were counted and the percentage computed. These percentages were categorized according to a criteria adapted from a previous study [52]; high similarity (more than 80%), moderate similarity (50–79%) and low similarity (less than 50%). This was done for 26 protein pairs. Protein pairs with low similarity were excluded from further analysis.

### Drug lead list

All approved drugs whose protein targets are similar to *P*. *falciparum* proteins were keyed alongside their respective proteins in a spreadsheet. Drugs that are applied topically, nutraceuticals and protein based drugs (e.g. insulin) were excluded from the drug lead list because they are less likely to be used as antimalarial drugs considering their physicochemical properties. Duplicate drug entries were also eliminated. A literature search was carried out at PubMed and Google Scholar to identify which drugs in the lead list had undergone *in vitro* testing for antimalarial activity. The literature search was done by searching the name of the drug with either “malaria”, “malaria *in vitro* testing” or “*plasmodium falciparum*”. Drugs that had been tested but their IC_50_ not documented were considered as not tested.

### *In vitro* drug susceptibility assays

A representative group of 10 drugs from those predicted to have activity were selected for *in vitro* testing of antiplasmodial activity. Selection of the drugs was based on their approved uses; two antileukemic drugs (cladribine and clofarabine), three anticancer drugs (oxaliplatin, dasatinib and irinotecan), an antibiotic (levofloxacin), one antidipsotropic (daidzin), an antiasmatic (zafirlukast), an immunosuppressive (tacrolimus) and one used to treat erectile dysfunction (tadafil). Seven of the drugs tested had no documentation of prior *in vitro* testing and three did. Details of their uses, putative drug targets, predicted *P*. *falciparum* drug targets (with druggability indices) and percentage of shared conserved regions between the two proteins are shown in Table 1. Chloroquine, dihydroartemisinin and mefloquine were tested (as reference standards) alongside the candidate drugs. All candidate drugs were bought from Sigma-Aldrich, while reference drugs were provided by World Wide Antimalarial Resistance Network (WWARN) Reference Standards Programme. The *P*. *falciparum* 3D7 parasites were obtained from Kenya Medical Research Institute-Walter Reed Project (KEMRI-WRP), Kisumu.

**Table 1:** Approved uses, putative targets, predicted *P*. *falciparum* targets, druggability indices and percentage of shared conserved residues of candidate drugs tested for *in vitro* antiplasmodial activity.

Drug	Indication (approved use)	Putative target (UNIPROT ID)	*P*. *falciparum* target (NCBI acc. No.)	% of shared conserved residues	Druggability index
Cladribine	Hairy cell leukemia	Adenosine deaminase (P00813)	Adenosine deaminase (XP_001347573.1)	55%	1
Daidzin	Anti-dipsotropic	ATP-binding cassette sub-family G member 2 (Q9UNQ0)	ABC transporter (XP_001348418.1)	51%	0.5
Zafirlukast	Asthma	ATP-binding cassette sub-family G member 2 (Q9UNQ0)	ABC transporter (XP_001348418.1)	51%	0.5
Levofloxacin	Antibacterial	DNA topoisomerase 2-alpha (P11388)	DNA topoisomerase II (XP_001348490.1)	61%	0.8
Dasatinib	Anticancer	ATP-binding cassette sub-family G member 2 (Q9UNQ0)	ABC transporter (XP_001348418.1)	51%	0.5
Clofarabine	Antileukemia	ATP-binding cassette sub-family G member 2 (Q9UNQ0)	ABC transporter (XP_001348418.1)	51%	0.5
Tacrolimus	Organ transplant	NA*	FK506-binding protein (FKBP)-type peptidyl-propyl isomerase (XP_001350859.1)	NA*	0.6
Irinotecan	Colorectal cancer	ATP-binding cassette sub-family G member 2 (Q9UNQ0)	ABC transporter (XP_001348418.1)	51%	0.5
Oxaliplatin	Colorectal cancer	ATP-binding cassette sub-family G member 2 (Q9UNQ0)	ABC transporter (XP_001348418.1)	51%	0.5
Tadafil	Erectile dysfunction	CGMP-specific 3',5'-cyclic phosphodiesterase (O76074)	3',5'-cyclic nucleotide phosphodiesterase (XP_001349954.1)	> 5%	-

*Predicted *P*. *falciparum* protein targets obtained from STITCH database (e.g. FK506-binding protein) did not have corresponding putative targets for comparison hence were not analyzed by the ConSurf server

The drugs were assayed using a non-radioisotopic assay technique described by Smilkstein and co-workers [8] with modifications [67, 68]. Reference clone chloroquine-sensitive (3D7) were cultured as described by Johnson and colleagues [67]. Drugs and compounds were dissolved in 99.5% dimethylsulfoxide (DMSO) (Sigma-Aldrich) and diluted in complete Roswell Park Memorial Institute 1640 series of Cell Culture Medium (RPMI 1640) prepared as described by Akala et al. [69]. The basic culture medium was prepared from 10.4 g RPMI 1640 powder (Invitrogen, Inc.) augmented with 2 g glucose (Sigma Inc.) and 5.95 g of HEPES (Sigma Inc.) dissolved to homogeneity in one litre of de-ionized water and sterilized with a 0.2 μM filter. Complete RPMI 1640 media (used for all parasite cultures and drug dilutions) consisted of basic RPMI 1640 media with 10% (vol/vol), human ABO pooled plasma, 3.2% (vol/vol) sodium bicarbonate (Thermo Fisher Scientific Inc.) and 4.0 μg/ml hypoxanthine (Sigma Inc.). Complete RPMI 1640 media was stored at 4°C and used within two weeks. Concurrently, two-fold serial dilutions of chloroquine (0.977 to 2,000 ng/ml), mefloquine (0.244 to 500 ng/ml), dihydroartemisinin (0.098 to 200 ng/ml) and drug candidates (24.414 to 50,000 ng/ml) were prepared on a 96-well plate, such that the amount of DMSO was equal to or less than 0.0875%. The 12 doses for each drug were added to wells in a row across the 96-well drug plate. *In vitro* drug testing was initiated when the culture-adapted *P*. *falciparum* at 5% hematocrit with greater than 3% parasitemia were adjusted to 2% hematocrit and 0.5% parasitemia, then added on to the plate containing a dose range of drugs and incubated in gas mixture comprising 5% CO_2_, 5% O_2_, and 90% N_2_ at 37°C. Each drug was tested in three biological replicates. The assay was terminated after 72 hours with SYBR Green dye added in lysis buffer and kept in the dark for 24 hours as described by Cheruiyot et al. [68]. The fluorescence intensity was measured from the bottom of the plate with a GENios Plus plate reader, with excitation wavelengths of 485 nm, emission wavelengths of 535 nm, gain set at 60 and number of flashes set at 10. Parasite growth inhibition was quantified using GraphPad Prism software version 5.02 from GraphPad Software Inc. CA, USA as described by Johnson et al. [67] and presented as mean ± standard deviation.

## Results

**Fig 2:**
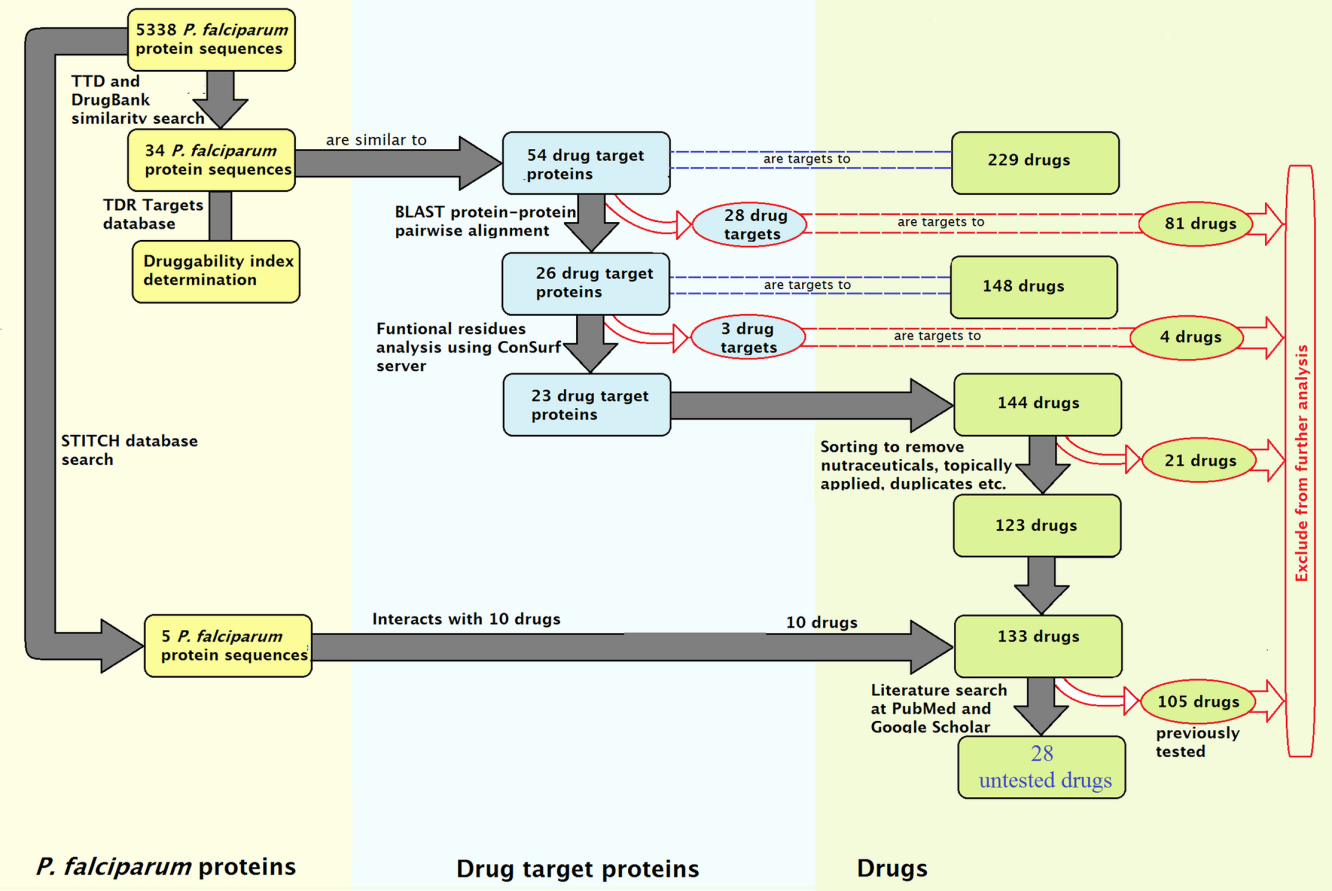
Steps in the chemogenomics repositioning workflow and their corresponding results. The yellow boxes represent *P*. *falciparum* sequences, drug targets are shown in blue boxes and drugs in green. Excluded drugs and proteins target have red box outlines.

A summary of results for each step in the study is shown in Fig 2 with more details in consecutive sections.

### *P*. *falciparum* proteome

A total of 5,338 protein sequences were obtained from NCBI RefSeq database [53]. This number represents all the *P*. *falciparum* protein sequences in RefSeq release 75.

### Identification of putative drug targets using drug databases

Each of the 5,338 protein sequences was used to search DrugBank, TTD and STITCH 4.0 databases. Using an E value cutoff of 1e-20 for DrugBank and TTD, 54 approved drug targets were identified to be similar to 34 *P*. *falciparum* possible targets. The 54 drug targets were associated with 229 approved drugs, the full list is shown in S1 Table. Using a minimum confidence score 0.7 for STITCH 4.0, 10 drugs were predicted to interact with 5 *P*. *falciparum* proteins. It is worth noting that some query results were similar in many of the searches while drugs that had multiple targets appeared more than once in the results.

### Druggability index

Druggability indices of 34 predicted *P*. *falciparum* drug targets obtained from TDR database are shown in Table 2. The least druggable drug target in the study had a druggability index of 0.3 while five had an index of 1. Eight proteins did not have their druggability indices in TDR database.

**Table 2:** Druggability indices of predicted *P*. *falciparum* drug targets.

*P*. *Falciparum* protein name	NCBI accession number	Druggability index
INOSINE-5'-MONOPHOSPHATE DEHYDROGENASE	XP_001352079.1	1
TUBULIN BETA CHAIN	XP_001347369.1	1
ADENOSINE DEAMINASE	XP_001347573.1	1
ADP/ATP TRANSPORTER ON ADENYLATE TRANSLOCASE	XP_001347650.1	1
RIBONUCLEOTIDE REDUCTASE SMALL SUBUNIT	XP_001348226.1	1
MO15-RELATED PROTEIN KINASE	XP_001347426.1	0.9
DNA TOPOISOMERASE II	XP_001348490.1	0.8
HISTONE DEACETYLASE	XP_001352127.1	0.8
RIBONUCLEOTIDE REDUCTASE SMALL SUBUNIT	XP_001347439.2	0.8
HISTONE DEACETYLASE	XP_001347363.1	0.7
CGMP-DEPENDENT PROTEIN KINASE	XP_001348520.1	0.7
M1-FAMILY ALANYL AMINOPEPTIDASE	XP_001349846.1	0.6
FKBP TYPE PEPTIDYL-PROPYL ISOMERASE	XP_001350859.1	0.6
SERINE/THREONINE PROTEIN PHOSPHATASE	XP_001348315.1	0.6
PREPROCATHEPSIN C PRECURSOR	XP_001350862.2	0.6
ACETYL-COA ACETYLTRANSFERASE	XP_001348658.1	0.6
CYCLIC NUCLEOTIDE PHOSPHODIESTERASE	XP_001348846.2	0.5
ABC TRANSPORTER	XP_001348418.1	0.5
CALCIUM/CALMODULIN-DEPENDENT PROTEIN KINASE	XP_001348401.2	0.5
GUANYLYL CYCLASE	XP_001348065.1	0.5
TRANSPORTER	XP_001349605.2	0.5
STROMAL-PROCESSING PEPTIDASE	XP_001348556.2	0.5
ACYL COA:DIACYLGLYCEROL ACYLTRANSFERASE	XP_001351293.1	0.4
GUANYLYL CYCLASE BETA	XP_001350316.2	0.4
CYSTEINE PROTEINASE FALCIPAIN-1	XP_001348727.1	0.3
FLAVODOXIN-LIKE PROTEIN	XP_002808949.1	0.3
CENTRIN-3	XP_001347555.2	Not available
CGMP-SPECIFIC PHOSPHODIESTERASE	XP_001350504.2	Not available
DELTA-AMINOLEVULINIC ACID DEHYDRATASE	XP_001348555.1	Not available
ORNITHINE AMINOTRANSFERASE	XP_966078.1	Not available
RNA BINDING PROTEIN	XP_001347313.1	Not available
3',5'-CYCLIC NUCLEOTIDE PHOSPHODIESTERASE	XP_001349954.1	Not available
HEAT SHOCK PROTEIN 110	XP_001349002.1	Not available
FERROCHELATASE	XP_001350360.2	Not available

### Determination and comparison of functional regions

A protein-protein pairwise alignment performed between the *P*. *falciparum* proteins and their corresponding homologous drug targets revealed 26 out of 54 protein pairs had at least 80% query coverage. The 26 were selected for ConSurf server analysis while the rest were excluded. Comparison of the functional amino acids residues revealed eight protein pairs had high similarity (more than 80%), 11 had a moderate similarity (50–79%) and three low similarity (less than 50%). Those with low similarity were excluded from further analysis.

### Drug lead list

The successful 23 putative target proteins were associated with 144 drugs in DrugBank and TTD databases while 5 *P*. *falciparum* proteins were predicted to interact with 10 drugs in the STITCH database. This made a total of 154 drugs that were predicted to have antimalarial activity in this study. Out of the 154 drugs, 21 drugs are either applied topically, duplicates, protein based or pure elements, these were eliminated from the drug lead list. A literature search revealed 105 drugs out of the remaining 133 to have been previously tested for antimalarial activity. The IC_50_ of some of the tested drugs are shown in Table 3 while those that had not been tested previously are summarized in Table 4.

**Table 3:** IC_50_ values of some previously tested approved drugs that were predicted to have antiplasmodial activity.

Predicted *P*. *falciparum* target, its druggability index and % of shared conserved residues	Drug	Indication	Antimalarial activity (IC_50_*)	References
**Predicted *P*. *falciparum* target**: ABC transporter (XP_001348418.1) **Putative target**: ABC sub-family G member 2 (Q9UNQ0) **Druggability index:** 0.5 **% of shared conserved residues**: 51%	Dactinomycin	Antibiotic	0.0009 μM	[48]
	Cisplatin	Anticancer	0.021 μM	[70]
	Cyclosporine	Immuno-suppressant	0.032 μM	[71]
	Docetaxel	Anticancer	0.01 μM	[72]
	Doxorubicin	Antibiotic	0.21 μM	[48]
	Ivermectin	Antiparasitic	9.1 μM	[73]
	Lamivudine	Antiretroviral	> 50 μM	[74]
	Saquinavir	Antiretroviral	5 μM	[74]
	Vincristine	Anticancer	0.0021 μM	[75]
**Predicted *P*. *falciparum* target:** DNA Topoisomerase II (XP_001348490.1) **Putative target:** DNA topoisomerase 2-alpha (P11388) **Druggability index:** 0.8 **% of shared conserved residues**: 61%	Amsacrine	Cutaneous T Cell Lymphoma	0.1 to 2.8 μM	[76]
	Ciprofloxacin	Antibiotic	20 μM	[77]
	Enoxacin	Antibiotic	120 μM	[75]
	Fleroxacin	Antibiotic	94 μM	[75]
	Lovastatin	Hypolipidemic	>200 μM	[78]
	Norfloxacin	Antibiotic	55 μM	[75]
	Ofloxacin	Antibiotic	180 μM	[75]
	Sparfloxacin	Antibiotic	140 μM	[75]
	Trovafloxacin	Antibiotic	27 μM	[79]
	Dactinomycin	Antibiotic	0.0009 μM	[48]
**Predicted *P*. *falciparum* target:** Histone deacetylase (XP_001347363.1) **Putative target:** Histone deacetylase (Q13547) **Druggability index:** 0.8 **% of shared conserved residues:** 99%	Trichostatin A	Antifungal, Antibiotic	—	[80]
	Valproic Acid	Epilepsy And Seizures Treatment	210 μM	[75]
	Vorinostat	Cutaneous T Cell Lymphoma	0.12 μM	[49]
**Predicted *P*. *falciparum* target:** IMP dehydrogenase (XP_001352079.1) **Putative target:** IMP dehydrogenase 1 (P20839.2) **Druggability index: 1** **% of shared conserved residues** NA	Azathioprine	Immunosuppressant	≥ 1 μM	[81]
**Predicted *P*. *falciparum* target:** IMP dehydrogenase (XP_001352079.1) **Putative target:** IMP dehydrogenase 2 (P12268) **Druggability index:** 1 **% of shared conserved residues:** 79%	Mycophenolic Acid	Immunosuppressant	5.4 μM	[82]
**Predicted *P*. *falciparum* target:** Serine/threonine protein phosphatase (XP_001348315.1) **Putative target:** Serine/threonine-protein phosphatase PP1-alpha catalytic subunit (P08129) **Druggability index:** 0.6 **% of shared conserved residues**: 99%	Cantharidin	Warts	3 μM	[83]
**Predicted *P*. *falciparum* target:** Tubulin beta chain (XP_001347369.1) **Putative target:** Tubulin beta-4B chain (P68371) **Druggability index:** 1 **% of shared conserved residues:** 99%	Albendazole	Anthelmintic	2 μM	[48]
	Vinblastine	Anticancer	0.0072 μM	[75]
	Vindesine	Anticancer	0.006 μM	[75]
	Vincristine	Anticancer	0.0021 μM	[75]
**Predicted *P*. *falciparum* target:** Cyclic nucleotide phosphodiesterase (XP_001348846.2) **Putative target:** cAMP-specific 3',5'-cyclic phosphodiesterase 4A (P27815) **Druggability index:** 0.5	Dipyridamole	Anticoagulants	0.03 μM	[84]
**Predicted *P*. *falciparum* target:** Adenosine deaminase (XP_001347573.1) **Putative target:** Adenosine deaminase (P00813) **Druggability index:** 1 **% of shared conserved residues:** 55%	Dipyridamole	Anticoagulants	0.03 μM	[84]
**Predicted *P*. *falciparum* target:** Centrin-3 (XP_001347555.2) **Putative target:** Calmodulin (P62158) **Druggability index:** NA **% of shared conserved residues:** 76%	Trifluoperazine	Antipsychotic, Antiemetic	0.47 μM	[85]
**Predicted *P*. *falciparum* target:** Calcium/calmodulin-dependent protein kinase (XP_001348401.2) **Putative target:** CaM kinase II subunit gamma (Q13555) **Druggability index:** 0.5 **% of shared conserved residues:** 47%	Bosutinib	Chronic Myelogenous Leukemia (CML)	0.22 μM	[48]

*All IC_50_ values are converted to μM

**Table 4:** Details of approved drugs predicted to target *P*. *falciparum* proteins that have not been tested.

Drug	UNIPROT ID of putative target	NCBI ACCESSION NUMBER OF *P*. *FALCIPARUM* TARGET	CONSURF RESULTS	DRUGGABILITY OF *P*. *FALCIPARUM* TARGET
Cladribine	P00813	XP_001347573.1	55%	1
Fludarabine	P00813	XP_001347573.1	55%	1
Epirubicin	P11388	XP_001348490.1	61%	0.8
Finafloxacin	P11388	XP_001348490.1	61%	0.8
Palbociclib	P11802	XP_001347426.1	54%	0.9
Capridine-beta	P24941	XP_001347426.1	70%	0.9
Motexafin gadolinium	P31350	XP_001347439.2	60%	0.8
Aprindine	P62158	XP_001347555.2	76%	-
Venlafaxine	Q9UNQ0	XP_001348418.1	51%	0.5
Oxaliplatin	Q9UNQ0	XP_001348418.1	51%	0.5
Zafirlukast	Q9UNQ0	XP_001348418.1	51%	0.5
Clofarabine	Q9UNQ0	XP_001348418.1	51%	0.5
Sumatriptan	Q9UNQ0	XP_001348418.1	51%	0.5
Irinotecan	Q9UNQ0	XP_001348418.1	51%	0.5
Buprenorphine	Q9UNQ0	XP_001348418.1	51%	0.5
Idelalisib	Q9UNQ0	XP_001348418.1	51%	0.5
Cobicistat	Q9UNQ0	XP_001348418.1	51%	0.5
Lenvatinib	Q9UNQ0	XP_001348418.1	51%	0.5
Daclatasvir	Q9UNQ0	XP_001348418.1	51%	0.5
Osimertinib	Q9UNQ0	XP_001348418.1	51%	0.5
Pitavastatin	Q9UNQ0	XP_001348418.1	51%	0.5
Rilpivirine	Q9UNQ0	XP_001348418.1	51%	0.5
Apixaban	Q9UNQ0	XP_001348418.1	51%	0.5
Vandetanib	Q9UNQ0	XP_001348418.1	51%	0.5
Biricodar dicitrate	Q9UNQ0	XP_001348418.1	51%	0.5
Daidzin	Q9UNQ0	XP_001348418.1	51%	0.5
Cabazitaxel	Q9UNQ0	XP_001348418.1	51%	0.5
Tacrolimus	STITCH	XP_001350859.1	-	0.6

### *In vitro* drug susceptibility assays

*In vitro* drug susceptibility tests carried out on ten drugs showed eight with activity within the concentration ranges used. Their mean IC_50_ are shown in Table 5 while the three biological replicate readings are displayed in S3 Table.

**Table 5:** *In vitro* activities of drugs tested against *P*. *falciparum* 3D7 strain.

Drugs	Mean IC_50_ ± SD (μM)
Tadafil	23.29 ± 2.41
Irinotecan	14.35 ± 1.70
Levofloxacin	40.10 ± 5.28
Oxaliplatin	1.16 ± 0.10
Clofarabine	48.95 ± 2.03
Tacrolimus	4.52 ± 0.08
Cladribine	96.02 ± 16.43
Dasatinib	8.60 ± 2.22
**Reference drugs**	
Chloroquine	0.0116 ± 0.0004
Dihydroartemisinin	0.0026 ± 0.0001
Mefloquine	0.0395 ± 0.0063

The table shows the mean IC_50_ values and the standard deviation for the drugs in μM as tested in this study. Each drug was tested in three replicates.

## Discussion

This study was based on the principle that if a *P*. *falciparum* protein is similar to a confirmed drug target, by inference the drug in question would have a similar effect on the *P*. *falciparum* protein. Using the full proteome of *P*. *falciparum* to do a target-similarity search in drug databases, the study predicted 133 approved drugs could target 34 *P*. *falciparum* proteins. A literature search showed 105 of the 133 drugs to have been previously tested against *P*. *falciparum*, showing a strong research interest in repositioning approved drugs. Most of the drugs that were previously tested did show activity, validating the use of this approach in drug repositioning. *In vitro* drug susceptibility tests were done on 10 drugs that were predicted to have antiplasmodial activity. Seven drugs out of the 10 tested did show significant activity with IC_50_ ranging from 1 μM to 50 μM, these include levofloxacin, dasatinib, clofarabine, tacrolimus, irinotecan, oxaliplatin and tadafil. The drugs that did show activity should be considered for further evaluation and development. The drug-target associations predicted in this study could also explain possible mechanisms of action of drugs that were active, this information could be used to develop more potent antimalarial drugs.

### Target-similarity search

This study was based on the assumption that two proteins that similar are by inference likely to share ligands. However, it is important to note that a drug could target a protein/pathway other than the one it is predicted to inhibit. These “off targets” are not uncommon since many drugs have been documented to have multiple targets. In addition, high sequence similarity between two proteins does not necessarily mean they would have similar biological roles. Homologs could have different biological functions but still meet the similarity thresholds used in this study. Nevertheless, the similarity of prospective protein targets to known drug targets has been used as the basis for past repositioning attempts [51,52,86]. This could also explain why most of the drugs predicted to have antimalarial activity in this study are already tested (Table 3).

The whole proteome of *P*. *falciparum* was used to perform the similarity search, therefore drugs that could target all stages of the parasite’s life cycle were considered in this study. Most drug development efforts focus on erythrocytic stages because they cause symptoms of the disease and are easier to manipulate in the laboratory. In fact, current antimalarial drugs were discovered on the basis of their activity against the red blood cell stage parasite. Developing drugs targeting the exo-erythrocytic and sporogonic cycles are increasingly drawing interest [9,18–20] because all stages of the parasite need to be targeted if malaria is to be eliminated [21]. The effect of the current antimalarials on all the life cycle stages of *Plasmodium* has also been studied [87].

The sequence similarity search in both TTD and DrugBank databases found duplication of some results with several *P*. *falciparum* proteins picking similar targets. For instance, many of the drug target searches yielded same kinases with low E values. It is interesting to note that kinases are one of the most common classes of proteins investigated as drugs targets in *P*. *falciparum* [37–42]. The similarity in the BLAST output could be attributed to the paralogous nature of many *P*. *falciparum* proteins and their orthology to many putative drug targets. Among the drug targets that showed up frequently in the output results, Plasmodium merozoite surface protein 1 (PMSP1) was the most common. Being a *Plasmodium* protein itself, it is expected to be similar to many paralogous *P*. *falciparum* proteins we used as queries. Notwithstanding, the similarity of PMSP1 protein to several of *P*. *falciparum* proteins is worth investigating further. It is also worth noting that PMSP1 antigen is not a target of any approved or experimental drug, rather it is being investigated for vaccines in clinical trials [88]. Other examples of protein targets that showed up frequently include troponin C, heat shock protein 40, calmodulin, centromeric protein E and Rho-associated protein kinase 1.

### ATP-binding cassette transporters

The *P*. *falciparum* ATP-binding cassette (ABC) transporter (NCBI accession number Q9UNQ0) was predicted in this study to be a potential target to five drugs that were tested *in vitro*; dasatinib, clofarabine, irinotecan, daidzin and zafirlukast. The putative target of these drugs is the human ABC sub-family G member 2 (NCBI accession number Q9UNQ0), also known as breast cancer resistance protein (BRCP) and multidrug resistant protein 1 (MRP1). The BRCP is classified among multidrug resistant proteins (MRPs) because of its role in drug resistance and treatment failures in trypanosomatid, apicomplexan and amitochondriate parasites of clinical significance [89,90]. It is believed to cause treatment failures by actively translocating a wide range of structurally and functionally diverse amphipathic compounds across cellular membranes [91]. The ABC transporters have been implicated with high IC_50_ values in response to chloroquine and quinine in *P*. *falciparum* field isolates [92]. Members the MRP family of proteins should also be considered as potential targets for antimalarial drugs because of the vital role they have been shown to play in blood stage multiplication of the *Plasmodium* species [93]. ABC transporters also have been considered as targets for antibacterial vaccines and chemotherapies because of the part they play in transporting molecules across membranes [94]. The *P*. *falciparum* ABC transporter has moderate druggability (D index of 0.5). It also shares 51% of conserved amino acid residues and an E value of 2e-61 when aligned with the human homolog drug target (BRCP) using protein-protein BLAST, suggesting a strong similarity.

Dasatinib has been approved for treatment of chronic myelogenous leukemia (CML) and is currently being evaluated for use in treating other cancers [95–97]. It is widely documented that dasatinib acts by inhibiting a range of tyrosine-protein kinases [98–102], these include Bcr-Abl, Lck and Src family of tyrosine kinases. Dasatinib also has other targets including the human MRP 1 [103,104]. Previous *in vitro* drug susceptibility assays on *P*. *falciparum* have shown dasatinib to have an IC_50_ of >10 μM [105] compared to 8.599 ± 2.222 μM determined in this study. Other drugs that were tested that could target the *P*. *falciparum* ABC transporter are antileukemia clofarabine (48.95 ± 2.032μM) and anticancer drug irinotecan (14.35 ± 1.7 μM). Daidzin (an anti-dipsotropic) and zafirlukast (an antiasthmatic) did not show any activity at the concentration ranges used.

### Adenosine deaminase

The target similarity approach used by this study also predicted *P*. *falciparum* adenosine deaminase, ADA (XP_001347573.1) to be a target of cladribine, dipyridamole, fludarabine and pentostatin. (S1 Table). This protein has an E value of 2e-29 when aligned with its homologous drug target (P00813.3) and they share 55% of conserved residues. Additionally, the high druggability of *P*. *falciparum* ADA (D index of 1) makes it a strong drug target candidate. *P*. *falciparum* ADA is essential to the survival of the parasite since the parasite is unable to synthesize purine bases and hence relies on purine salvage and purine recycling to meet its purine needs. *P*. *falciparum* ADA is unique because it catalyzes the deamination of both adenosine and 5‘-methylthioadenosine while the human form cannot deaminate the latter [106]. Sriram et al. [107] used a bioinformatics approach to show how quinine and primaquine could bind to the ADA protein. 5‘-methylthio coformycins have also been shown to inhibit the *P*. *falciparum* ADA without inhibition of its human homolog [106]. Examples of 5‘-methylthio coformycins that have been tested against 3D7 clone of *P*. *falciparum* include 5'-Methylthio-immucillin-H (MT-ImmH) and immucillin-H (ImmH) which have IC_50_ values of 63 nM and 50 nM respectively [108]. This is comparable to dipyridamole’s IC_50_ of 30 nM [84] which is also predicted by this study to target the *P*. *falciparum* ADA. *In vitro* tests carried out in this study also showed cladribine to have an IC_50_ of 96.02 μM which is much higher than that of MT-ImmH, ImmH and dipyridamole. This could probably be attributed cladribine having weaker inhibition of ADA (assuming it is the only target) or other experimental factors. Nevertheless, an antimalarial that could effectively block the parasite’s purine salvage pathway would be efficient in inhibiting the parasite’s growth.

### DNA topoisomerases

Levofloxacin, like most broad-spectrum fluoroquinolones, acts by inhibiting two type II DNA topoisomerase enzymes in bacteria; DNA gyrase and topoisomerase IV [109,110]. Levofloxacin was predicted in this study to inhibit the activity of *P*. *falciparum’*s DNA topoisomerase II (XP_001348490.1). The *P*. *falciparum* DNA topoisomerase II has an E value of 0.0 when aligned to two distinct homologous drug targets; DNA topoisomerase 2-alpha (P11388) and DNA topoisomerase 2-beta (Q02880) suggesting a high similarity. Besides, the *P*. *falciparum* DNA topoisomerase II has a D index of 0.8 and shares 61% functional amino acid residues with the drug target DNA topoisomerase 2-alpha. DNA topoisomerases enzymes are involved in overwinding or underwinding of DNA during DNA replication and transcription, hence are considered essential to the survival of many organisms including *P*. *falciparum*. Several types of DNA topoisomerases have been characterized and are classified into two major classes depending on how they change the topology of DNA: topoisomerase I and topoisomerase II [111]. Garcia-Estrada et al. [112] proposed DNA topoisomerases as attractive drug targets because of structural differences between host and apicomplexan isoforms, differential expression patterns as well as lack of orthologous topoisomerases in mammals since there are no apicoplast DNA gyrases in mammals. Levofloxacin showed activity against *P*. *falciparum* 3D7 with an IC_50_ of 14.17 μg/ml in this study. A total of 30 approved drugs were predicted to target the *P*. *falciparum* DNA topoisomerase II (S1 Table). Examples include moxifloxacin, ciprofloxacin, lucanthone and epirubicin. Camptothecin, a potent DNA topoisomerase I inhibitor has been shown to inhibit nucleic acid biosynthesis in *P*. *falciparum* suggesting that it could also be targeting the *Plasmodium* homolog [113]. Nevertheless, camptothecin was predicted in this study to inhibit the *Plasmodium*’s ABC transporter (S1 Table) because of its similarity to the MRP1 [114]. Unfortunately, camptothecin cannot be used as an antimalarial considering its toxicity.

### Histone deacytalase

*P*. *falciparum* histone deacytalase, HDAC (XP_001352127.1) shares 99% of functional amino acid residues with drug target histone deacetylase 2 (Q92769) and 85% with histone deacetylase 1 (Q13547). The *Plasmodium* homologue has an E value 0.0 when aligned with both the histone deacetylase proteins, suggesting a strong similarity. Therefore, the *Plasmodium* HDAC could be targeted by drugs such as vorinostat (recommended treatment for T cell lymphoma), valproic acid (used for epilepsy treatment) and trichostatin A (an antifungal and antibacterial). All the three drugs have been shown to target the histone deacetylase 2 (Q92769). *Plasmodium* HDAC has high druggability (a D index of 0.8), making it an attractive antimalarial drug target. A recent study assessed the role of HDAC inhibitors in impeding the growth of *P*. *falciparum* both *in vivo* and *in vitro* [115]. Vorinostat has displayed high *in vitro* antimalarial activity with an IC_50_ of 0.12 μM [49] while valproic acid had 209.34 μM [75]. HDAC inhibitors have also been investigated as drugs for a range of other diseases such as trypanosomiasis, schistosomiasis, leishmaniasis, toxoplasmosis, HIV/AIDS and even cancer [116]. Apicidin, a novel fungal metabolite, has been documented as an inhibitor of HDAC in apicomplexan parasites including malaria [117]. The main challenge about the *in vivo* use of many HDAC inhibitors is that their zinc-binding hydroxamate group is broken down resulting in lose activity [115].

### Inosine 5'-monophosphate dehydrogenase

Inosine 5'-monophosphate dehydrogenase (IMPDH) plays a key role in catalyzing the first committed step of guanosine 5'-monophosphate biosynthesis, an essential pathway in *P*. *falciparum*. The *P*. *falciparum* IMPDH (XP_001352079.1) has high druggability (D index of 0.8), an E value of 8e-169 when aligned with its putative drug target homologue IMPDH 2 (P12268.2) with which it also shares 79% of conserved amino acid residues. It also has an E value of 4e-173 when aligned with another homologous drug target, the IMPDH 1 (P20839.2). Both these drugs targets are human isoforms. The IMPDH is an attractive target for many therapeutic interventions since most parasites depend on the salvage pathway due to their inability to synthesize purine nucleotides *de novo*. Inhibitors of IMPDH, ribavirin and mycophenolic acid (both target IMPDH 1 and IMPDH 2) have been used as immunosuppressives, antivirals and anticancer drugs with few side effects to host cells [118–120]. Nevertheless, little has been done concerning their application in treating microorganisms [121]. This study predicted mycophenic acid to inhibit the *P*. *falciparum* IMPDH and it has been shown to be active against *P*. *falciparum* with an IC_50_ of 5.4 μM [82].

### FK506-binding protein-12

Tacrolimus (FK506) is used to lower the risk of organ rejection after an allogenic organ transplant. It brings about its immunosuppressive activity by binding to FK506-binding protein-12 (FKBP-12) to form a complex that inhibits calcineurin, consequently preventing both T-lymphocyte activation and interleukin-2 transcription [122]. Tacrolimus has been shown by Bell and colleagues [71] to inhibit the growth of *P*. *falciparum in vitro*, with an IC_50_ of 1.9 μM compared to 4.521 ± 0.083 μM established in this study. The study by Bell et al. could not ascertain the mechanism of action considering they could not detect FKBPs in *P*. *falciparum* extracts at the time of the study. However, the genome sequence of *P*. *falciparum* [22] revealed that it does have a 35-kDa FKBP (PfFKBP35). Though the function of PfFKBP35 is still unknown, the presence of tetratricopeptide repeat motifs suggests it may be involved in transporting and modulating the function of other proteins in the parasite [123]. Bao and colleagues [124] showed tacrolimus could prevent the development of cerebral malaria in *Plasmodium berghei* ANKA-infected mice though it failed to clear the parasites at the concentrations used. This could mean PfFKBP35 and any other protein tacrolimus targets don’t play a critical role in the survival of the parasite.

### Challenges and limitations

The validation approach used in this study assumes that the drugs that have shown activity against *Plasmodium* would be inhibiting the predicted *P*. *falciparum* protein targets. This might not be necessarily true because drugs have been shown to inhibit parasite growth by acting on targets other than the proteins they were expected to considering many drugs interact with several targets. An example of such a drug is dasatinib which targets several tyrosine-protein kinases [98–102], platelet-derived growth factor receptor beta [125], dimethylaniline monooxygenase 3 [126], signal transducer and activator of transcription 5B [127], ABC transporters [103] and a number of cytochrome P450 proteins [128]. Such drugs could bring their inhibitory activity either through concerted efforts of multiple targets or through a few targets that are involved in crucial pathways. This was not factored in the validation process. Nonetheless, multi-target drugs have been documented to be more effective than single-target ones [129] and less prone to drug resistance [130]. Furthermore, targeting different proteins/pathways is the basis for drug combination therapies [4,131].

Based on the low number of similar drug targets detected during the similarity search, it is probable that the parameters used in this study to filter results may have been too stringent. These parameters include an E value of 1e-20 in sequence similarity, a query coverage of 80% in protein-protein pairwise alignment and a minimum of 50% of shared conserved amino residues. For instance, bosutinib has been documented to have high antimalarial activity with an IC_50_ of 0.22 μM [48]. On the other hand, bosutinib’s predicted *P*. *falciparum* target, the calcium/calmodulin-dependent protein kinase (XP_001348401.2) shares 47% of functional amino acids residues with its homologous putative target, the calcium/calmodulin-dependent protein kinase type II subunit gamma (Q13555). This is below the threshold of 50% used in this study hence was eliminated at ConSurf server analysis stage. Out of the 5,338 *P*. *falciparum* protein sequences, only 34 possible drug targets met the inclusion criteria used in the DrugBank and TTD databases search and five in the STITCH database. Nevertheless, this had the benefit of increasing the likelihood of finding drug targets that were similar to the *P*. *falciparum* proteins hence increase the odds of discovering drugs with antimalarial effects. This also reduced the number of protein targets and drugs that would be analyzed in downstream processes. However, bosutinib represents many drugs (and their possible targets) that could otherwise have been identified by this approach but were not due to the rigorous inclusion criteria used.

## Conclusion

With the urgent need to develop new antimalarial drugs to counteract the increasing resistance to current ones, novel *P*. *falciparum* pathways should be targeted in the search for the next generation of antimalarial drugs. Repositioning of approved drugs offers such a strategy since most of these drugs have their putative targets documented. This information could be used to identifying approved drugs with antimalarial activity and reveal possible proteins and pathways that could be targeted in the search for new antimalarials. Furthermore, this approach can also be implemented in the search for drugs that are active against pathogens other than *P*. *falciparum*. The predicted drugs that did show significant *in vitro* activity against *P*. *falciparum* need be investigated further for antimalarial treatment.

## Supporting information

S1 Table Complete predicted drug list with their putative and predicted *P*. *falciparum* target. Those obtained from STITCH don’t have putative drug targets since they were associated directly with their corresponding *P*. *falciparum* proteins.
(XLSX)

S2 Table ConSurf results; amino acid conservation analysis spreadsheet. The table details the residue variety in% (across 150 homologs used in Consurf server analysis) for each position in the query sequence (drug target NP_001277159.1). Each column shows the% for that amino-acid, position it is found in the MSA. Non-standard amino acid residues are shown under column 'OTHER'.
(CSV)

S3 Table IC_50_ values (μM) of drugs tested *in vitro* on *P*. *falciparum* 3D7 strain (three replicates each). (DOCX)
