# Supplementary material for: Target-similarity search using Plasmodium falciparum proteome identifies approved drugs with anti-malarial activity and their possible targets
Source: PLoS One. 2017 Oct 31;12(10):e0186364. doi: 10.1371/journal.pone.0186364 (PMC5663372; doi:10.1371/journal.pone.0186364)
Supplement: S3 Table — (DOCX) [file pone.0186364.s003.docx]

|  | IC_50_ in µM | | | |
| --- | --- | --- | --- | --- |
| REPLICATES | **1** | **2** | **3** | **Mean ± SD** |
| Tadafil | 25.711 | 20.886 | 23.279 | **23.290 ± 2.413** |
| Irinotecan | 12.942 | 16.237 | 13.861 | **14.350 ± 1.700** |
| Levofloxacin | 44.260 | 34.165 | 41.871 | **40.100 ± 5.276** |
| Oxaliplatin | 1.154 | 1.057 | 1.258 | **1.156 ± 0.1003** |
| Clofarabine | 46.892 | 50.955 | 48.999 | **48.950 ± 2.032** |
| Tacrolimus | 4.6143 | 4.497 | 4.453 | **4.521 ± 0.083** |
| Cladribine | 94.782 | 113.026 | 80.245 | **96.020 ± 16.430** |
| Dasatinib | 7.955 | 11.071 | 6.770 | **8.599 ± 2.222** |
| Reference drugs | | | | |
| Chloroquine | 0.011326 | 0.011289 | 0.012055 | **0.01156 ± 0.000432** |
| Dihydroartemisinin | 0.002619 | 0.002741 | 0.002559 | **0.00264 ± 0.0000929** |
| Mefloquine | 0.033702 | 0.038698 | 0.046126 | **0.03951 ± 0.006251** |
